# Competitive Particle Swarm Optimization for Multi-Category Text Feature Selection

**DOI:** 10.3390/e21060602

**Published:** 2019-06-18

**Authors:** Jaesung Lee, Jaegyun Park, Hae-Cheon Kim, Dae-Won Kim

**Affiliations:** School of Computer Science and Engineering, Chung-Ang University, 221, Heukseok-Dong, Dongjak-Gu, Seoul 06974, Korea

**Keywords:** multi-label text categorization, feature selection, hybrid search, evolutionary algorithm, particle swarm optimization

## Abstract

Multi-label feature selection is an important task for text categorization. This is because it enables learning algorithms to focus on essential features that foreshadow relevant categories, thereby improving the accuracy of text categorization. Recent studies have considered the hybridization of evolutionary feature wrappers and filters to enhance the evolutionary search process. However, the relative effectiveness of feature subset searches of evolutionary and feature filter operators has not been considered. This results in degenerated final feature subsets. In this paper, we propose a novel hybridization approach based on competition between the operators. This enables the proposed algorithm to apply each operator selectively and modify the feature subset according to its relative effectiveness, unlike conventional methods. The experimental results on 16 text datasets verify that the proposed method is superior to conventional methods.

## 1. Introduction

Text categorization involves the identification of the categories associated with specified documents [1,2,3,4]. According to the presence or frequency of words within a document, the so-called bag-of-words model represents each document as a word vector [5]. Each word vector is then assigned to multiple categories because, in general, a document is relevant to multiple sub-concepts [6,7,8]. Text datasets are composed of a large number of words. However, not all the words are useful for solving the associated problem. Irrelevant and redundant words can confound a learning algorithm, deteriorating the performance of text categorization [9]. To resolve these issues, conventional methods have attempted to identify a subset of important words by discarding unnecessary ones prior to text categorization [10,11,12,13]. Thus, multi-label feature selection can be an effective preprocessing step for improving the accuracy of text categorization.

Given a set of word features $F=\{f_{1},\ldots ,f_{d}\}$, multi-label feature selection involves the identification of a subset S⊂F or a solution composed of n≪d features that are significantly relevant to the label set $L=\{l_{1}\ldots ,l_{\left|L\right|}\}$. To solve this task, conventional approaches use feature wrappers and filters. At the risk of selecting ineffective features for the learning algorithm to be used subsequently, filters can rapidly identify a feature subset that is mostly composed of important features based on the intrinsic properties of the data [14]. In contrast, wrappers directly determine the superiority of candidate feature subsets by using a specific learning algorithm. Moreover, they generally outperform the filters in terms of the learning performance [10]. Notwithstanding their essential differences, devising an effective search method is important in both approaches. This is because the algorithm must locate the final feature subset from a vast search space specified by thousands of word features.

As an effective search method for feature wrappers, population-based evolutionary algorithms are frequently used in conventional studies because of their stochastic global search capability [15]. These evolutionary algorithms evaluate the fitness of a feature subset based on the categorization performance of the learning algorithm. Furthermore, an evolutionary operator such as a mutation operator modifies the feature subset. Moreover, recent studies have reported that the search capability of an evolutionary algorithm can be further improved through hybridization with a filter [16,17]. Specifically, the feature filter operator can rapidly improve the feature subset by considering only the intrinsic properties of the data, particularly when the solution is overwhelmed by unnecessary features [18].

To achieve an effective hybrid search, the fitness of the feature subset modified by an evolutionary or filter operator must be improved. However, the fitness of a feature subset is not always improved after modification. This is because the evolutionary operator exhibits random properties, and the filter operator is independent of the fitness evaluation function [17,19,20,21]. If the fitness is not improved after modification by each operator, the modified feature subset is discarded. Thereby, computations performed to evaluate the fitness are wasted. A preferred hybrid search is one in which the modification of a feature subset by each operator always improves the fitness, thus avoiding wastage of computation. If an algorithm can ascertain the fitness after modification by each operator without evaluating the feature subset, it can decide in advance which operator in the feature subset is to be modified. However, this is unfeasible in practice [20]. The second-best option may be a method that estimates the relative effectiveness of each operator based on the fitness of the feature subset already computed in the previous iteration and decides which operator to apply. According to our experiment, although selective engagement of operators can significantly increase the effectiveness of a hybrid search, less attention has been paid to this aspect in recent studies.

To overcome the problems described above, we devise a competitive particle swarm optimization (PSO) algorithm. Unlike conventional PSOs, the proposed method applies each operator selectively based on a novel process for estimating the effectiveness of each operator for each particle. As a result, the particles can be separated into two groups depending on which operator is to be applied in the next iteration. Then, based on the fitness of the particles in each group, a tournament is run. Its results decide which operators will be applied in the next iteration by changing their memberships. Consequently, the proposed method competitively engages each operator in a feature subset search through a fitness-based tournament of the feature subset in each iteration. Our contributions are as follows:

- We proposed a novel competitive particle swarm optimization for multi-label feature selection problem by employing an information-theoretic multi-label feature filter as a filter operator.
- To selectively apply the evolutionary and filter operators, we proposed a new process for estimating their relative effectiveness based on the fitness-based tournament of the feature subset in each iteration.
- To demonstrate the superiority of the information-theoretic measure for improving the search capability, we employed an information-theory-based feature filter and a frequency-based feature filter simultaneously and conducted an in-depth analysis.

Our experiments revealed that the proposed method outperformed conventional methods. It indicates the effectiveness of the proposed estimation process and information-theoretic feature filter operator.

## 2. Related Work

In the field of text categorization, feature selection is a crucial task because the feature space is generally high-dimensional. Conventional feature selection methods can be largely categorized into feature filters and feature wrappers. Feature filter methods assess the importance of features using a score function such as the $\chi^{2}$ statistic, information gain, or mutual information [14]. The top-*n* features containing the highest scores are then selected. Uysal and Gunal [22] proposed a distinguishing feature selector that investigates the relationship between the absence or presence of a word within a document and the correct label for that document. Rehman et al. [23] proposed a normalized difference measure to remedy the problem of a balanced accuracy measure. It omits the relative document frequency in the classes. Tang et al. [24] proposed a maximum discrimination method based on a new measure for multiple distributions, namely the Jeffreys-multi-hypothesis divergence. However, these methods exhibit limited categorization accuracy because they do not interact with the subsequent learning algorithm.

In contrast, feature wrapper methods evaluate the discriminative power of feature subsets based on a specific learning algorithm and select the best feature subset. Among feature wrapper methods, population-based evolutionary algorithms are widely used for text feature selection owing to their stochastic global search capability. Aghdam et al. [25] applied ant colony optimization to text feature selection. Meanwhile, Lin et al. [26] proposed an improved cat swarm optimization algorithm to reduce the computation time of their originally proposed method. Lu et al. [27] demonstrated the enhanced performance of PSO based on a functional constriction factor and an inertia weight. However, unlike feature filters, these methods generally require significant computational resources for identifying a high-quality feature subset because of their randomized mechanism [28].

To resolve this issue, recent studies have considered hybrid approaches that combine an evolutionary feature wrapper with a filter. These hybrid methods can be categorized into two types according to how the filter operator is applied. One type applies the filter operator to initialize the population of the evolutionary algorithm during the initialization step. For example, Lu and Chen [21] initialized the candidate feature subsets of a small world algorithm using the $\chi^{2}$ statistic and information gain. Meanwhile, Mafarja and Mirjalili [18] initialized ants in a binary ant lion optimizer using a quick reduct and an approximate entropy reduct based on rough set theory. Although this approach involves the algorithm starting its search from a region exhibiting potential, the algorithm can be deficient in diversity, resulting in premature convergence. In addition, these algorithms can fail to refine the final feature subset because the filter operator is not engaged in the final stage of the search.

The second type of hybrid approach applies the filter operator to modify the feature subset in each iteration during the search process. Ghareb et al. [16] proposed an enhanced genetic algorithm by modifying the crossover and mutation operations by using the ranks of features obtained from six filter methods. Lee et al. [29] proposed an exploration operation that uses a filter to select important features from among those not selected by a genetic operator. Then, a new feature subset is generated. Moradi and Gholampour [30] constructed an enhanced binary PSO using correlation information. Meanwhile, Mafarja and Mirjalili [31] improved the whale optimization algorithm using simulated annealing for the local search. Dong et al. [19] enhanced the genetic algorithm using granular information to address feature selection in high-dimensional data with a low sample size. Zhou et al. [32] proposed a hybrid search that adjusts the influence of the feature filter according to the degree of convergence. However, these methods exhibit limited performance because the evolutionary and filter operators are not engaged selectively. Table 1 presents a brief summary of conventional feature-selection approaches.

## 3. Proposed Method

### 3.1. Preliminary

To design a competitive hybrid search, we selected PSO as an evolutionary algorithm because it has been demonstrated to be effective in many applications including feature selection [33,34,35,36]. PSO techniques can be classified into continuous PSO and binary PSO. In the former, the population is composed of real numbers. Meanwhile, in binary PSO, the population is composed of zeros and ones. In this study, we considered continuous PSO because binary PSO exhibits potential limitations such as the update of particles based solely on the velocity [36].

In continuous PSO for feature selection, the population of particles is known as a swarm. The location of a particle with *d* elements can be regarded as a probability vector each of whose elements is the probability that the corresponding feature is selected. The location of a particle is described as follows:

(1) $$C_{i}=\left[C_{i}\left(1\right),C_{i}\left(2\right),\ldots ,C_{i}\left(j\right),\ldots ,C_{i}\left(d\right)\right],$$

where $C_{i}$ is the *i*th particle in particle group *C* and $C_{i}\left(j\right)$ is the probability that the *j*th feature is selected when the feature subset is generated from $C_{i}$ in our study. In the initialization step, the elements of each location are initialized as real numbers obtained at random from the uniform distribution [0,1].

To find feature subsets exhibiting potential, the particle locations are iteratively updated as follows:

(2) $$C_{i}\leftarrow C_{i}+V_{i},$$

where $V_{i}$ is the velocity vector of the *i*th particle; it refers to the magnitude and direction with which the particle moves across the search space. In the initialization step, the velocity of each particle is initialized randomly as a real number obtained from the uniform distribution [−1,1]. The velocity is calculated as follows:

(3) $$V_{i}\leftarrow wV_{i}+c_{1}r_{1}\left(P_{i}-C_{i}\right)+c_{2}r_{2}\left(G-C_{i}\right),$$

where $P_{i}$ is the so-called “personal best” and denotes the best location identified so far by the *i*th particle. *G* is the “global best” and denotes the best location identified so far by the swarm. Specifically, the best locations are selected according to a fitness value obtained by the specific learning algorithm. The inertia weight *w* controls the influence of the previous velocities on the present velocity. Here, $c_{1}$ and $c_{2}$ are acceleration constants, and $r_{1}$ and $r_{2}$ are random values uniformly distributed in [0,1]. Additionally, the velocity is limited to a maximum velocity $v_{\max}$ such that $\forall i,j:|V_{i}\left(j\right)|<v_{\max}$. In this study, these user-defined parameters are set based on conventional studies, to w=0.7298, $c_{1}=c_{2}=1.49618$, and $v_{\max}=0.6$ [36].

### 3.2. Motivation and Approach

**Figure 1:**
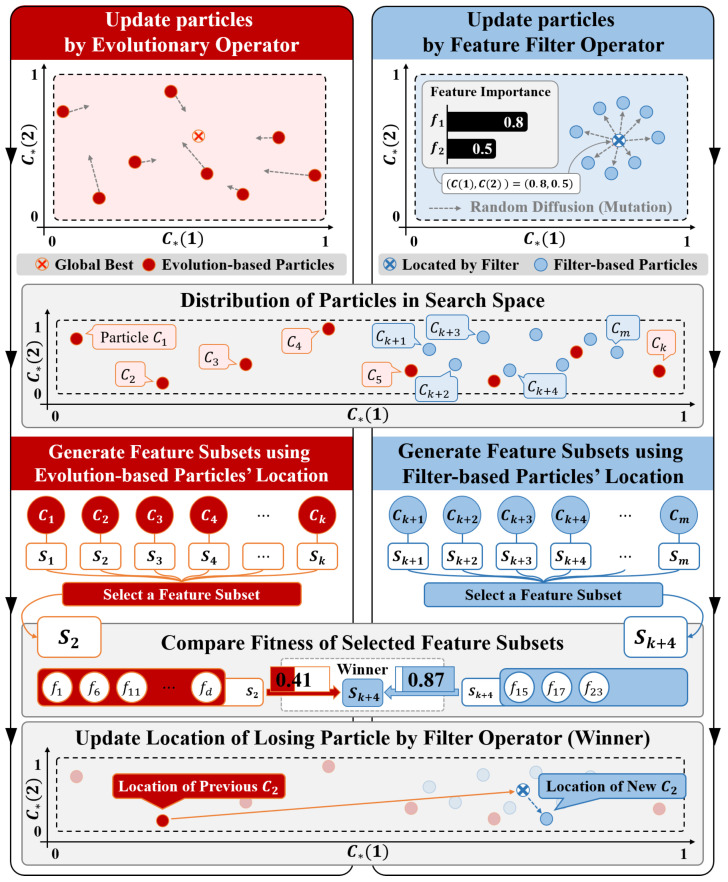
Schematic overview of the competitive particle swarm optimization.

We enhance the performance of a hybrid search for multi-label text feature selection by implementing competitive engagement of the evolutionary and filter operators according to their relative effectiveness. To estimate their relative effectiveness and implement competitive engagement, each operator needs to modify the particles independently in each iteration. Therefore, we separate the particles into small groups depending on which operator is applied in the next iteration, i.e., evolution-based and filter-based particle groups.  Figure 1 shows a schematic overview of the proposed algorithm.

First, we design the evolution-based particle group based on conventional PSO. In the initialization step, the evolution-based particles are assigned real numbers obtained at random from the uniform distribution [0,1]. During the search process, feature subsets are generated using the particle locations (similarly as in conventional PSO), as described in Section 3.1. In addition, they are updated according to Equations (2) and (3) by the evolutionary operator. Secondly, the filter-based particle group is initialized and updated by the filter operator using a score vector obtained from a score function corresponding to the filter. The elements of the score vector are the importances of the features.

During the search process, the algorithm updates the membership of the losing particles with that of the winning particle, according to the tournament results based on the fitness value. This is shown in  Figure 1. For example, if a filter-based particle wins, the filter operator’s search is regarded to be more effective than that of the evolutionary operator in the previous iteration. Thus, the algorithm applies the filter operator to the losing evolution-based particle in the next iteration. This procedure is repeated until the parameterized resources are exhausted.

### 3.3. Competitive Particle Swarm Optimization

Although multiple filter-based operators can be employed in our proposed method, for simplicity, we outline the pseudocode of the proposed method in a case in which only one filter is used. This is illustrated in Algorithm 1. The terms used to describe the algorithm are summarized in Table 2. In the initialization step (Line 3), the algorithm generates evolution-based and filter-based particles with Algorithm 2. On Lines 4 and 5, each particle generates a feature subset using its location. This feature subset is evaluated by a fitness function, in which the obtained fitness value $E_{c},E_{f}$ denotes the learning performance of the text categorization. In this pseudocode, a high fitness value indicates that the corresponding particle displays good fitness. Additionally, because $m_{c}+m_{f}$ particles are being evaluated, there are $m_{c}+m_{f}$ fitness function calls (FFCs) on Line 6. The number of FFCs is generally used as a stopping criterion [20].

**Algorithm 1** Competitive particle swarm optimization.
1:**input:**$m_{c},m_{f},v$; ▹ The number of particles for each group $m_{c},m_{f}$, the maximum number of FFCs *v*2:**output:***S*;                              ▹ the final feature subset *S*3:[C,F]←**initialization**$\left(m_{c},m_{f}\right)$;             ▹ initialize particles using **Algorithm 2**4:$[S_{c},S_{f}]\leftarrow$**generate** subsets based on C,F;              ▹ use locations of particles5:$[E_{c},E_{f}]\leftarrow$**evaluate** subsets $S_{c},S_{f}$;           ▹ evaluate subsets using fitness function6:$u\leftarrow m_{c}+m_{f}$;                      ▹ set *u* to the number of whole particles7:
**while** u<v **do**
8:    **update** *C* using Equations (2) and (3);               ▹ update locations of particles9:    $[S_{c},S_{f}]\leftarrow$ **generate** subsets based on C,F;10:    $[E_{c},E_{f}]\leftarrow$ **evaluate** subsets $S_{c},S_{f}$;11:    $u\leftarrow u+m_{c}+m_{f}$;12:    $[C,F,m_{c},m_{f}]\leftarrow$ **competition**$\left(C,F,E_{c},E_{f},m_{c},m_{f}\right)$;          ▹ use **Algorithm 3**13:    S← the best feature subset so far;14:
**end while**



**Algorithm 2** Initialization function.
1:**input:**$m_{c},m_{f}$;              ▹ The number of particles for each group $m_{c}$, $m_{f}$2:**output:**C,F;                           ▹ initialized particles3:**for**k=1 to $m_{c}$ **do**4:    **for** j=1 to *d* **do**5:        $C_{k}\left(j\right)\leftarrow$ **sample** from U(0,1);              ▹ use uniform distribution6:    **end for**7:
**end for**
8:X←**calculate** a score vector;             ▹ use score function of feature filter9:σ←**calculate** a standard deviation;                  ▹ use Equation (4)10:
$\sigma \leftarrow \sigma m_{f}$
11:**for**k=1 to $m_{f}$ **do**12:    **for** j=1 to *d* **do**13:        $F_{k}\left(j\right)\leftarrow$**sample** from $N(X,\sigma^{2})$;            ▹ use Gaussian distribution14:    **end for**15:
**end for**



After the initialization process, the evolution-based particles are updated by the evolutionary operator on Line 8. Moreover, all particles are evaluated by the fitness function on Lines 9 and 10. On Lines 12 and 13, the evolution- and filter-based particles compete. The losing particles are updated in the next iteration by the winning operator, according to the competition results from Algorithm 3. This procedure is repeated until the algorithm attains the maximum FFCs, denoted by *v*. The output of Algorithm 1 is the best feature subset obtained during the search process.

**Algorithm 3** Competition function.
1:**input:**$C,F,E_{c},E_{f},m_{c},m_{f}$;               ▹ fitness values for each group $E_{c},E_{f}$2:**output:**$C,F,m_{c},m_{f}$;                ▹ changed particles via competitions C,F3:$w_{c}\leftarrow 0$;4:$l_{c}\leftarrow 0$;5:**for**k=1 to $\min(\left[m_{c},m_{f}\right])$ **do**               ▹ set the number of competitions6:    **if** $\max\left(E_{c}\right)>\max\left(E_{f}\right)$ **then**7:        $w_{c}\leftarrow w_{c}+1$;              ▹ add one whenever evolution-based particle wins8:    **else if** $\max\left(E_{c}\right)<\max\left(E_{f}\right)$ **and** $m_{c}\neq l_{c}+1$ **then**9:        $l_{c}\leftarrow l_{c}+1$;               ▹ add one whenever evolution-based particle loses10:        $j\leftarrow \{j|\forall x\in \left\{\left[1,m_{f}\right]\cap Z\setminus j\right\},E_{f}\left(j\right)>E_{f}\left(x\right)\}$;11:        $E_{f}\left(j\right)\leftarrow -\infty$;               ▹ exclude winning particle at next competition12:    **end if**13:
**end for**
14:**for**k=1 to $w_{c}$ **do**15:    $j\leftarrow \{y|\forall x\in \left\{\left[1,m_{f}\right]\cap Z\setminus y\right\},E_{f}\left(y\right)<E_{f}\left(x\right)\}$;16:    **delete** $F_{j}$;                    ▹ delete the particle with low fitness value17:    $C_{end+1}\leftarrow$ a new particle;                    ▹ use uniform distribution18:
**end for**
19:**for**k=1 to $l_{c}$ **do**20:    $j\leftarrow \{y|\forall x\in \left\{\left[1,m_{c}\right]\cap Z\setminus y\right\},E_{c}\left(y\right)<E_{c}\left(x\right)\}$;21:    **delete** $C_{j}$;22:    $F_{end+1}\leftarrow$ a new particle;                ▹ use score vector for feature filter23:
**end for**
24:$m_{c}\leftarrow m_{c}+w_{c}-l_{c}$;25:$m_{f}\leftarrow m_{f}+l_{c}-w_{c}$;


Algorithm 2 presents the detailed procedure for initializing the particles. On Lines 3–7, the evolution- based particles are initialized. The score function associated with the filter then calculates a score vector to initialize the filter-based particles. If only one filter-based particle group is used, it is generated by the random diffusion of a score vector to maintain diversity within the group on Lines 9–15. Herein, random diffusion can be implemented by diffusing the score vector according to a Gaussian distribution. Therefore, the mean is set to the score vector, and the standard deviation is calculated as follows:

(4) $$\sigma =\frac{1}{d-1}\sum_{k=1}^{d-1}\left(X_{s}\left(k+1\right)-X_{s}\left(k\right)\right),$$

where $X_{s}$ is the score vector sorted in ascending order. This is calculated as the average score difference to prevent the diffusion from altering the ranking orders excessively. On Line 10, our algorithm multiplies the standard deviation by the number of filter-based particles to maintain diversity.

Algorithm 3 presents the detailed procedure of the competition between the evolution- and filter-based particles. On Line 5, the number of competitions is set to the minimum of the particle group sizes. On Lines 6–12, after each group has selected the particle with the maximum fitness value, the particles compete based on the fitness value. The competition results are stored as the number of winners and losers for the evolution-based particle group. Our algorithm prevents the number of evolution-based particles from becoming zero on Line 8. If a particle continues to win in the competition, all particles can converge to a particle by genetic drift [37]. To circumvent this, a winning particle is prevented from competing in the next competition, preventing any particle from continually winning (Lines 10 and 11). In our algorithm, the losing particles are updated by the winning operator in the next iteration on Lines 17 and 22.

Finally, we conducted a theoretical analysis of the time complexity of the proposed method. In the evolutionary search, each feature subset should be evaluated by the learning algorithm to obtain the fitness value. This involves complicated sub-procedures including a decision-making process for multiple categories and repetitive cross-validation to simulate realistic performance [38]. Thus, the maximum number of FFCs permitted can be used to represent the computational complexity of the proposed method, i.e., O(v).

### 3.4. Information-Theoretic Multi-Label Feature Filter Operator

The information theory is frequently used in conventional studies because of its capability to quantify the similarity between probability distributions. The information-theory-based feature filter methods generally evaluate the importance of features based on the joint entropy between each feature and labels. We selected the information-theoretic multi-label feature filter, namely quadratic programming-based multi-label feature selection [39], as a filter operator. This is because it has performed effectively in multi-label feature selection problems. It calculates a score vector based on a criterion that maximizes the dependency on labels and minimizes the redundancy among the features. Here, the score vector represents the importance of each feature.

Given a set of features $F=\{f_{1},\ldots ,f_{d}\}$ and label set $L=\{l_{1}\ldots ,l_{\left|L\right|}\}$, the score vector *X* is calculated by solving the following maximization problem:

(5) $$\max_{X}Q_{X}=\sum_{f_{i}\in F}\sum_{l_{j}\in L}I\left(f_{i};l_{j}\right)X\left(i\right)-\sum_{f_{i},f_{j}\in F}I\left(f_{i};f_{j}\right)X\left(i\right)X\left(j\right),$$

where I(a;b)=H(a)+H(b)−H(a,b) is the Shannon’s mutual information between the random variables *a* and *b*. $H\left(a\right)=-\sum_{i}p\left(a_{i}\right)\log_{2}\left(p\left(a_{i}\right)\right)$ is the joint entropy of the probability distributions p(a), p(b), and p(a,b). Specifically, the left-hand side implies dependency between each feature and multiple labels, and the right-hand side implies redundancy among features. In addition, the score vector *X* has the following constraints:

(6) $$X\left(1\right),X\left(2\right),\ldots ,X\left(d\right)\geq 0,\sum_{i=1}^{d}X\left(i\right)=1.$$

These constraints enable the consideration of the score vector *X* as a probability vector. Therefore, the score vector can be used as the particle’s location.

## 4. Experimental Results

### 4.1. Experimental Settings

We conducted experiments using 16 datasets from the RCV1 and Yahoo collections, which together comprise over 10,000 features. We used the top 2% and 5%, respectively, of the features with the highest document frequency because the categorization performance would not be affected significantly by the removal of features [40,41]. The datasets contain text data with multiple labels. Herein, the labels correspond to specific subjects related to the document. In the text data, each feature corresponds to the frequency of a word within the document. Table 3 presents the standard statistics for the multi-label datasets used in our experiments. The statistics include the number of patterns in the dataset |W|, number of features |F|, feature type, and number of labels |L|. In addition, the label cardinality Card represents the average number of labels for each pattern. Moreover, the label density Den is the label cardinality over the total number of labels. Furthermore, Distinct indicates the number of unique label subsets in *L*. The experiments conducted in this study included only text data.

We compared the proposed method with two hybrid-based feature selection methods and a PSO-based feature selection method: EGA + CDM [16], bALO-QR [18], and competitive swarm optimizer (CSO) [9], respectively. EGA + CDM combines an enhanced genetic algorithm (EGA) [16] with a class discriminating measure (CDM). bALO-QR initializes the ants in a binary ant lion optimizer (bALO) [42] using the quick reduct (QR). CSO is a PSO-based method that uses multiple swarm. For each method, the parameters were set to the values recommended in the original study, and a problem transformation enabled each label subset to be treated as a single class when calculating each filter algorithm. This is because these were designed to handle single-label datasets. To prevent bias, we set the maximum permissible FFCs to 300. The maximum number of selected features was set to 50. The population size was set to 30. To evaluate the quality of the feature subsets obtained by each method, we used the multi-label naive Bayes (MLNB) [43] and extreme learning machine for multi-label (ML-ELM) [44] classifier with the holdout cross-validation method. For each dataset, 80% of the data was selected for the training set. The remaining 20% was used as the test set. We performed each experiment 10 times and used the average value to represent the categorization performance of each feature selection method.

In the proposed method, to demonstrate the superiority of information-theoretic multi-label filter operator for improving search capability, we employed an additional frequency-based filter operator, namely a normalized difference measure [23]. In our experiments, it competes with the evolutionary operator as well as the information-theoretic filter operator. A comparison between the operators is described in Section 5. For three operators, we set the size of the corresponding particle group to 10.

To evaluate the performance of each feature selection method, we employed four evaluation metrics: Hamming loss, one-error, multi-label accuracy, and subset accuracy [45,46,47]. Let $T=\{\left(w_{i},\lambda_{i}\right)|1\leq i\leq |T|\}$ be a specified test set. Here, $\lambda_{i}\subseteq L$ is a correct label subset related to $w_{i}$. Given a test sample $w_{i}$, a predicted label set $Y_{i}\subseteq L$ is estimated by a classifier such as MLNB. In detail, a family |L| of functions $\left\{f_{1},f_{2},\ldots ,f_{\left|L\right|}\right\}$ is induced from the multi-label training examples. Here, each function $f_{k}$ determines the class membership of $l_{k}$ with respect to each instance, i.e., $Y_{i}=\{l_{k}|f_{k}\left(w_{i}\right)>\theta ,1\leq k\leq \left|L|\right\}$; moreover, θ is a predefined threshold. Using the correct label subsets and predicted label sets, we can compute the four metrics. The Hamming loss is defined as follows:

(7) $$hloss\left(T\right)=\frac{1}{\left|T\right|}\sum_{i=1}^{\left|T\right|}\frac{1}{\left|L\right|}\left|\lambda_{i}△Y_{i}\right|,$$

where △ denotes the symmetric difference between two sets. The one-error is defined as

(8) $$oneerr\left(T\right)=\frac{1}{\left|T\right|}\sum_{i=1}^{\left|T\right|}[\arg\max_{l_{k}\in L}f\left(w_{i}\right)\notin \lambda_{i}],$$

where [·] returns a value of one if the proposition stated in the brackets is true, and zero otherwise. The multi-label accuracy is calculated as

(9) $$mlacc\left(T\right)=\frac{1}{\left|T\right|}\sum_{i=1}^{\left|T\right|}\frac{|\lambda_{i}\cap Y_{i}|}{|\lambda_{i}\cup Y_{i}|}.$$

Finally, the subset accuracy is defined as

(10) $$setacc\left(T\right)=\frac{1}{\left|T\right|}\sum_{i=1}^{\left|T\right|}[\lambda_{i}=Y_{i}].$$

Higher values of the multi-label accuracy and subset accuracy and lower values of the Hamming loss and one-error indicate higher performance.

We conducted a statistical test to compare the proposed method to previous techniques. First, we employed the widely used Friedman test to compare multiple methods [38]. Based on the average rank for each method, the null hypothesis that all the methods perform equally well is either rejected or accepted. When the null hypothesis was rejected, we proceeded with a certain post-hoc test to analyze the relative performance among the methods being compared [38]. Thus, we employed the Bonferroni–Dunn test, which compares the difference between the average ranks of the proposed method and of another method [48]. For the Bonferroni–Dunn test, the performances of the proposed method and of the other methods are regarded as statistically similar if their average ranks over all datasets are within one critical difference (CD). In our experiments, the CD was 1.093 [38].

### 4.2. Comparison Results

**Figure 2:**
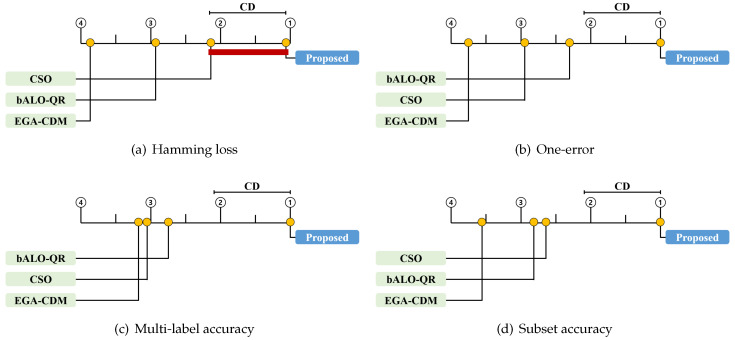
Bonferroni–Dunn test results of four comparison methods with four evaluation measures for MLNB.

**Figure 3:**
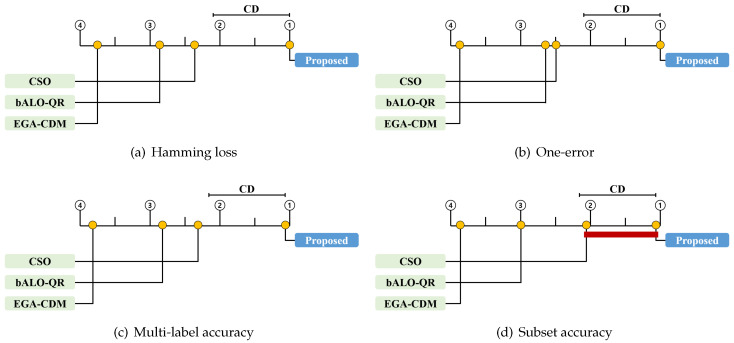
Bonferroni–Dunn test results of four comparison methods with four evaluation measures for ML-ELM.

Table 4, Table 5, Table 6, Table 7, Table 8, Table 9, Table 10 and Table 11 contain the experimental results for the proposed method and the other methods, on 16 multi-label text datasets. They are presented as the average performance, with the corresponding standard deviations. In Table 4, Table 5, Table 6 and Table 7 and Table 8, Table 9, Table 10 and Table 11, MLNB and ML-ELM, respectively, are used as classifiers. The highest performance is shown in bold font and indicated by a check mark. Finally, Table 12 and Table 13 contain the Friedman statistics and the corresponding critical values on each evaluation measure for each classifier. Here, we set the significance level α=0.05. In Figure 2 and Figure 3, the CD diagrams illustrate the relative performance of the proposed method and of other methods. Herein, the average rank of each method is marked along the upper axis, with the higher ranks placed on the right side of each subfigure. We also present the CD from the perspective of the proposed method above the graph. This implies that the methods within the range are not significantly different from each other [38]. Those for which the difference is not significant are connected by a thick line.

From the results in Table 4, Table 5, Table 6, Table 7, Table 8, Table 9, Table 10 and Table 11, it is evident that the proposed method outperformed the state-of-the-art feature selection methods for most of the multi-label text datasets. For MLNB, the proposed method achieved the highest performance on 94% of the datasets in terms of Hamming loss, and on all datasets in terms of one-error, multi-label accuracy, and subset accuracy. For ML-ELM, the proposed method achieved the highest performance on all datasets in terms of Hamming loss and one-error, and on 94% of the datasets in terms of multi-label accuracy and subset accuracy. Consequently, the proposed method consistently achieved the highest average rank in all the experiments. As shown in Figure 2, the proposed method significantly outperformed all other algorithms in terms of one-error, multi-label accuracy, and subset accuracy for MLNB. As shown in Figure 2a, the proposed method was significantly better than EGA-CDM and bALO-QR in terms of Hamming loss for MLNB. Figure 3 shows that the proposed method significantly outperformed all the other algorithms in terms of Hamming loss, one-error, and multi-label accuracy for ML-ELM. Figure 3d shows that the proposed method is significantly better than EGA-CDM and bALO-QR in terms of the subset accuracy for ML-ELM.

In summary, the experimental results demonstrate that the proposed method outperformed the three reference algorithms on 16 text datasets. Statistical tests verified that the proposed method was significantly superior to the other methods in terms of one-error, multi-label accuracy, and subset accuracy for MLNB and in terms of Hamming loss, one-error, and multi-label accuracy for ML-ELM.

## 5. Analysis for Engagement of the Evolutionary and Filter Operators

**Figure 4:**
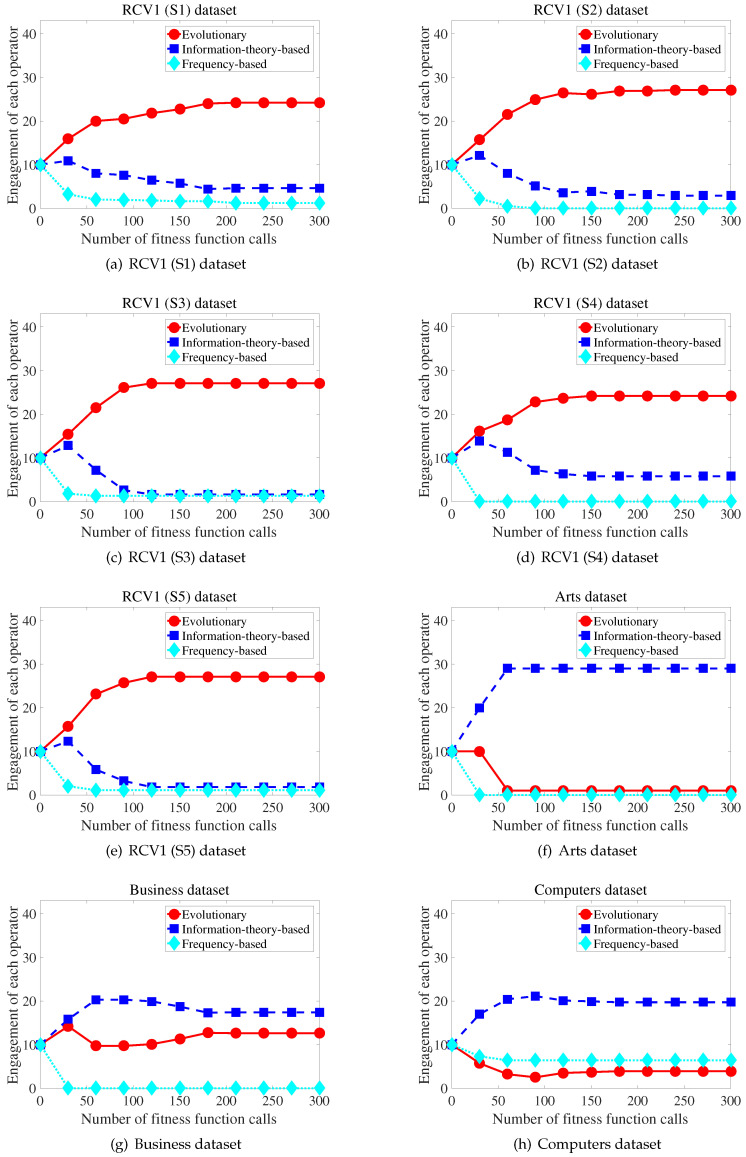

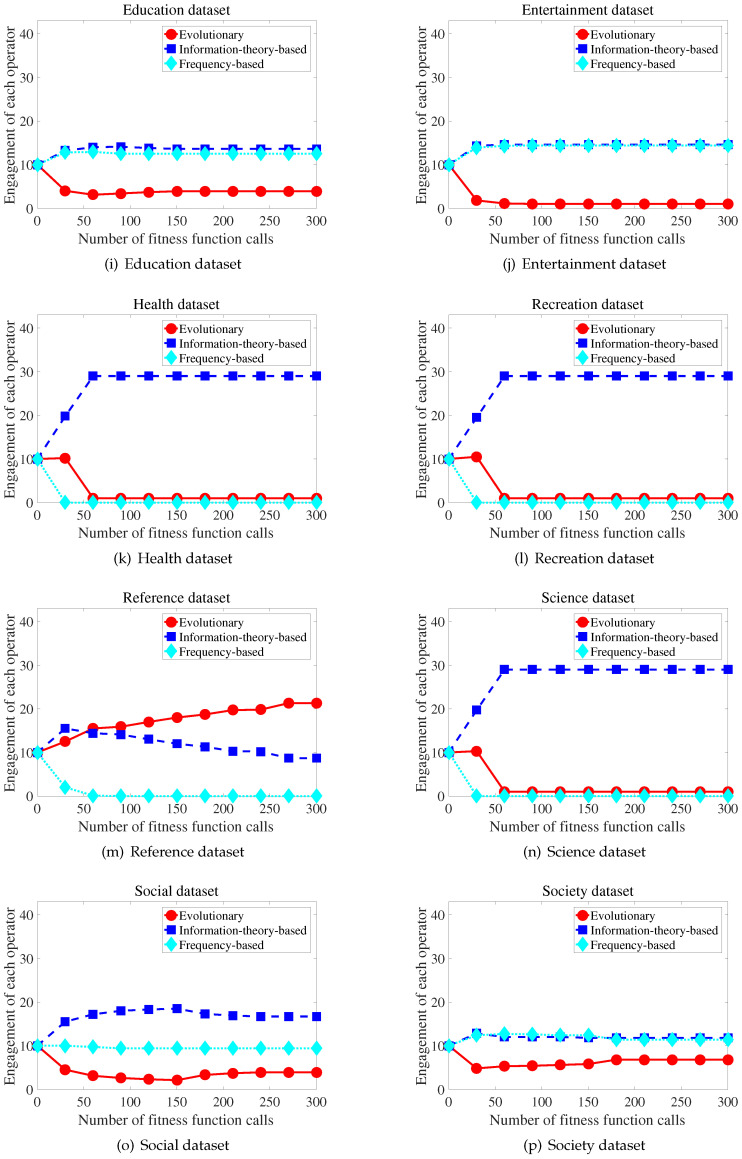
Competition results between particle groups for proposed method in terms of subset accuracy.

To describe the competition results for each iteration, Figure 4 shows the engagement of each operator during the search process. Here, each engagement is represented as the average across 10 experimental trials with MLNB. Specifically, the engagement refers to the number of times an evolutionary operator and two filter operators modify the particles in each iteration. As shown in Figure 4, the effectiveness of the operators could be varied according to the progress of search on a specified dataset. This indicates that the capability of evolutionary search and the performance of a filter method could vary. Such situations could be intensified in text applications owing to the sparsity of the data. Figure 4a–e shows that the filter operator could rapidly improve the particles on the RCV1 dataset in the early stages of the search process. Additionally, Figure 4m shows that the information-theory-based filter operator is more frequently engaged than the evolutionary operator in the early stages when the reference dataset was used. However, the information-theory-based filter operator was more frequently engaged than the evolutionary operator across the entire search process on the dataset in Figure 4f–l,n–p. Moreover, the frequency-based filter operator was more frequently engaged than the evolutionary operator on the dataset in Figure 4h–j,o–p. In addition, the information-theory-based filter operator was more frequently engaged than the frequency-based filter operator on 81% of the datasets in Figure 4. This demonstrates the superiority of the information-theoretic measure in improving the search capability.

This study was motivated by the consideration that competitive engagement via competition between the evolutionary and filter operators could improve the performance of the learning algorithm. To validate this, we conducted an additional experiment in which we compared the proposed method to a non-competitive reference algorithm. Specifically, in the initialization step, the particle groups were initialized as in the proposed method. However, the evolutionary and filter operators equally modified particles during the search process, unlike in the proposed method. We set the maximum permissible FFCs to 300 and the size of each particle group to 10, as stated in Section 4.

**Figure 5:**
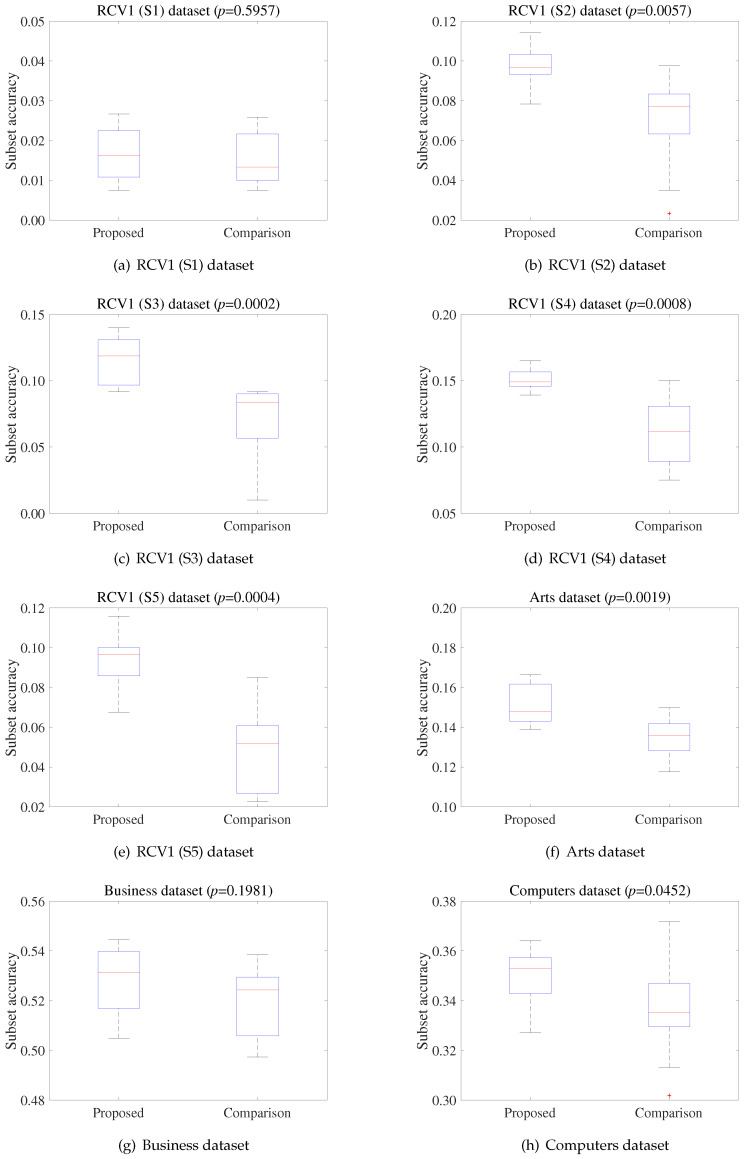

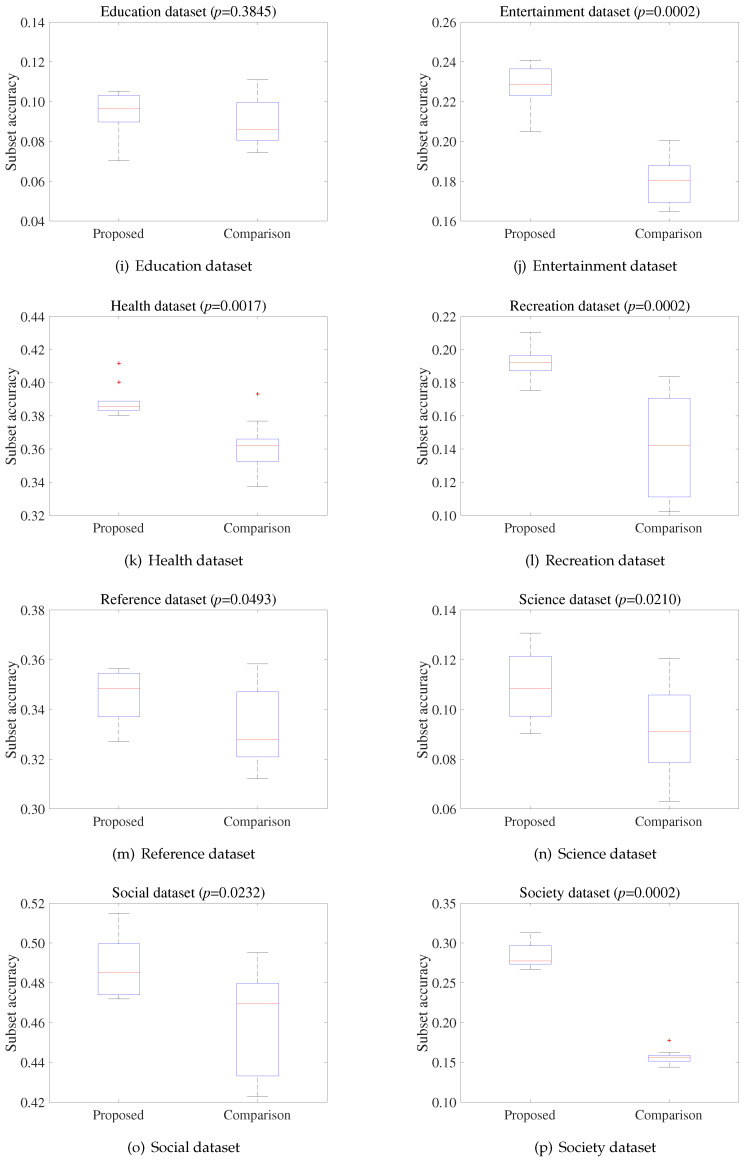
Comparison results between two methods in terms of subset accuracy.

Figure 5 compares the subset accuracy of the proposed and reference algorithm on 16 datasets using MLNB. In Figure 5, the vertical axis indicates the subset accuracy. To determine whether the two methods were statistically different for each dataset, we conducted a Wilcoxon rank sum test [49]. The corresponding *p*-values are shown in each subfigure. The test used the results from 10 repeated experiments on each dataset. As shown in Figure 5, the additional experiments demonstrate that the competitive engagement of the operators could improve the search capability.

## 6. Discussion

The main contribution of this study is the proposal of a new process for advance estimation of the relative effectiveness of the evolutionary and filter operators and their selective engagement in each iteration to improve the hybrid search. Our method compares the fitness of particles modified by each operator and determines the operator to be applied according to the results of the tournament.

The proposed method has the following advantages. By selectively applying each operator, our method can reduce the number of feature subsets that are discarded because of not having been improved after modification by each operator. That is, the method increases the number of times the fitness is improved. In addition, comparison of the effectiveness of each operator does not require additional computations. The proposed method permits more evolution-based particles to explore and exploit locations exhibiting potential, by increasing the engagement of the evolutionary operator when its effectiveness is higher than that of the filter operator. In the converse case, important features are selected with higher probabilities by increasing the engagement of the filter operator. In this regard, the proposed method may be more stable than conventional hybrid methods in the feature selection tasks. Other PSO variants (such as predator–prey PSO [50,51]) can be applied to our method. For example, multi-swarm PSO methods (such as competitive swarm optimizer [9]) can be applied by dividing evolution-based particles into multiple swarms. Similarly, other filter methods can be applied by using multiple filter operators.

In this study, a method for estimating the superiority of each operator is developed to improve the effectiveness of hybrid search. After the operator to be applied is selected, a new feature subset is created and evaluated. Thus, the proposed method selects between two feasible feature subsets: one is a feature subset modified by the evolutionary operator, and the other is a feature subset modified by the filter operator. This concept originated from the well-established informed best-first search [52], i.e., when the algorithm encounters several nodes to be visited, one is selected based on its potential, which is typically measured by a heuristic function or process. In our experiments, the superiority between the two operators was determined based on the fitness-based tournament. Furthermore, the effectiveness of our method was verified because the proposed method outperformed the reference algorithms.

Table 4, Table 5, Table 6, Table 7, Table 8, Table 9, Table 10 and Table 11 reveal that the proposed method outperformed three state-of-the-art methods. The results demonstrate that the proposed method is an effective feature selection method. Figure 5b–e shows that the proposed method exhibited higher exploration and exploitation capability than the reference algorithm as the search progressed. This is because, as shown in Figure 4, the evolution-based particles generated better feature subsets than the filter-based particles, on the RCV1 dataset. Furthermore, increasing the engagement of the evolutionary operator permitted more evolution-based particles to explore and exploit locations exhibiting potential. Figure 4j–l and Figure 5j–l show that, when the effectiveness of the filter operator was higher than that of the evolutionary operator, increasing the engagement of the filter operator aided in selecting the important features. Finally, the experimental results demonstrate that the competitive engagement of the operators could successfully improve the search performance.

## 7. Conclusions

Most conventional hybrid approaches for multi-label feature selection do not consider the relative effectiveness between the evolutionary and filter operators. In this study, we proposed a novel competitive hybrid approach for multi-label text feature selection aimed at improving the learning performance by selective engagement of the operators via competition. The experimental results and a statistical test verified that the proposed method significantly outperformed three state-of-the-art feature selection methods, on 16 multi-label text datasets.

Future research will focus on the applications for our approach. The proposed method was designed for multi-label text feature selection. However, it can be applied to other scenarios. The evolutionary feature wrapper and filters should be selected according to the specific application. We will study this issue further.

## Figures and Tables

**Table 1:** Brief summary of conventional feature selection approaches.

	Advantages	Disadvantages
Filter methods	Rapid identification of a feature subset	Lower performance than that of wrapper
Wrapper methods	High performance than that of filter	High complexity
Hybrid methods (first type)	To start in a region exhibiting potential	Premature convergence
Hybrid methods (second type)	Improved search capability	Randomized engagement of operator

**Table 2:** Notations used in the design of the proposed method.

Terms	Meanings
*C*	The evolution-based particle group
*F*	The filter-based particle group
$m_{c}$	The number of the evolution-based particles
$m_{f}$	The number of the filter-based particles
$E_{c}$	The fitness values for feature subsets generated from *C*
$E_{f}$	The fitness values for feature subsets generated from *F*
*u*	The number of spent fitness function calls (FFCs)
*v*	Maximum number of permitted FFCs
*S*	The best feature subset

**Table 3:** The standard statistics of multi-label text datasets.

Dataset	|W|	|F|	Type	|L|	Card.	Den.	Distinct.	Domain
RCV1 (S1)	6000	945	Numeric	101	2.880	0.029	1028	Text
RCV1 (S2)	6000	945	Numeric	101	2.634	0.026	954	Text
RCV1 (S3)	6000	945	Numeric	101	2.614	0.026	939	Text
RCV1 (S4)	6000	945	Numeric	101	2.484	0.025	816	Text
RCV1 (S5)	6000	945	Numeric	101	2.642	0.026	946	Text
Arts	7484	1157	Numeric	26	1.654	0.064	599	Text
Business	11,214	1096	Numeric	30	1.599	0.053	233	Text
Computers	12,444	1705	Numeric	33	1.507	0.046	428	Text
Education	12,030	1377	Numeric	33	1.463	0.044	511	Text
Entertainment	12,730	1600	Numeric	21	1.414	0.067	337	Text
Health	9205	1530	Numeric	32	1.644	0.051	335	Text
Recreation	12,828	1516	Numeric	22	1.429	0.065	530	Text
Reference	8027	1984	Numeric	33	1.174	0.036	275	Text
Science	6428	1859	Numeric	40	1.450	0.036	457	Text
Social	12,111	2618	Numeric	29	1.279	0.033	361	Text
Society	14,512	1590	Numeric	27	1.670	0.062	1054	Text

**Table 4:** Comparison results of four compared methods in terms of Hamming loss for MLNB (The highest performance is shown in bold font and indicated by a check mark).

Dataset	Proposed	EGA+CDM	bALO-QR	CSO
RCV1 (S1)	0.029 ± 0.001	0.030 ± 0.001	0.030 ± 0.000	**0.029 ± 0.001✓**
RCV1 (S2)	**0.027 ± 0.001✓**	0.028 ± 0.003	0.027 ± 0.001	0.027 ± 0.001
RCV1 (S3)	**0.026 ± 0.000✓**	0.027 ± 0.001	0.027 ± 0.001	0.026 ± 0.001
RCV1 (S4)	**0.024 ± 0.001✓**	0.025 ± 0.001	0.025 ± 0.001	0.024 ± 0.001
RCV1 (S5)	**0.026 ± 0.001✓**	0.028 ± 0.003	0.028 ± 0.001	0.026 ± 0.001
Arts	**0.061 ± 0.001✓**	0.067 ± 0.002	0.069 ± 0.002	0.066 ± 0.002
Business	**0.030 ± 0.001✓**	0.036 ± 0.004	0.034 ± 0.001	0.034 ± 0.002
Computers	**0.042 ± 0.002✓**	0.051 ± 0.004	0.046 ± 0.001	0.047 ± 0.001
Education	**0.043 ± 0.001✓**	0.048 ± 0.002	0.048 ± 0.002	0.047 ± 0.001
Entertainment	**0.059 ± 0.002✓**	0.069 ± 0.003	0.065 ± 0.001	0.065 ± 0.001
Health	**0.039 ± 0.001✓**	0.050 ± 0.003	0.047 ± 0.001	0.047 ± 0.002
Recreation	**0.058 ± 0.001✓**	0.070 ± 0.003	0.067 ± 0.002	0.065 ± 0.001
Reference	**0.031 ± 0.001✓**	0.040 ± 0.003	0.037 ± 0.002	0.037 ± 0.001
Science	**0.036 ± 0.001✓**	0.043 ± 0.003	0.042 ± 0.001	0.042 ± 0.001
Social	**0.026 ± 0.001✓**	0.042 ± 0.004	0.032 ± 0.002	0.032 ± 0.001
Society	**0.057 ± 0.001✓**	0.065 ± 0.004	0.064 ± 0.001	0.063 ± 0.001
Avg. Rank	**1.06✓**	3.88	2.94	2.13

**Table 5:** Comparison results of four compared methods in terms of one-error for MLNB (The highest performance is shown in bold font and indicated by a check mark).

Dataset	Proposed	EGA+CDM	bALO-QR	CSO
RCV1 (S1)	**0.573 ± 0.151✓**	0.637 ± 0.129	0.621 ± 0.134	0.648 ± 0.125
RCV1 (S2)	**0.513 ± 0.013✓**	0.654 ± 0.023	0.580 ± 0.015	0.599 ± 0.019
RCV1 (S3)	**0.609 ± 0.206✓**	0.718 ± 0.150	0.671 ± 0.174	0.683 ± 0.168
RCV1 (S4)	**0.591 ± 0.216✓**	0.696 ± 0.160	0.671 ± 0.175	0.672 ± 0.174
RCV1 (S5)	**0.603 ± 0.210✓**	0.695 ± 0.161	0.656 ± 0.182	0.652 ± 0.185
Arts	**0.649 ± 0.181✓**	0.712 ± 0.149	0.710 ± 0.149	0.712 ± 0.149
Business	**0.383 ± 0.410✓**	0.399 ± 0.409	0.398 ± 0.400	0.396 ± 0.406
Computers	**0.415 ± 0.009✓**	0.469 ± 0.006	0.445 ± 0.009	0.448 ± 0.007
Education	**0.598 ± 0.012✓**	0.661 ± 0.008	0.616 ± 0.020	0.639 ± 0.016
Entertainment	**0.536 ± 0.017✓**	0.605 ± 0.019	0.563 ± 0.015	0.586 ± 0.015
Health	**0.726 ± 0.342✓**	0.774 ± 0.282	0.764 ± 0.300	0.778 ± 0.238
Recreation	**0.553 ± 0.011✓**	0.739 ± 0.013	0.675 ± 0.013	0.675 ± 0.011
Reference	**0.690 ± 0.262✓**	0.715 ± 0.243	0.718 ± 0.241	0.715 ± 0.243
Science	**0.630 ± 0.024✓**	0.707 ± 0.018	0.696 ± 0.027	0.696 ± 0.023
Social	**0.439 ± 0.197✓**	0.571 ± 0.152	0.472 ± 0.186	0.490 ± 0.179
Society	**0.447 ± 0.014✓**	0.510 ± 0.017	0.489 ± 0.019	0.479 ± 0.016
Avg. Rank	**1.00✓**	3.75	2.31	2.94

**Table 6:** Comparison results of four compared methods in terms of Multi-label accuracy for MLNB (The highest performance is shown in bold font and indicated by a check mark).

Dataset	Proposed	EGA+CDM	bALO-QR	CSO
RCV1 (S1)	**0.198 ± 0.010✓**	0.176 ± 0.011	0.168 ± 0.013	0.124 ± 0.013
RCV1 (S2)	**0.243 ± 0.013✓**	0.177 ± 0.011	0.179 ± 0.014	0.157 ± 0.018
RCV1 (S3)	**0.227 ± 0.018✓**	0.161 ± 0.004	0.178 ± 0.019	0.168 ± 0.014
RCV1 (S4)	**0.267 ± 0.016✓**	0.170 ± 0.007	0.192 ± 0.014	0.183 ± 0.019
RCV1 (S5)	**0.234 ± 0.013✓**	0.187 ± 0.009	0.191 ± 0.016	0.165 ± 0.012
Arts	**0.195 ± 0.012✓**	0.094 ± 0.008	0.099 ± 0.008	0.106 ± 0.012
Business	**0.680 ± 0.010✓**	0.662 ± 0.009	0.654 ± 0.008	0.656 ± 0.011
Computers	**0.424 ± 0.007✓**	0.369 ± 0.010	0.388 ± 0.006	0.391 ± 0.008
Education	**0.122 ± 0.010✓**	0.075 ± 0.008	0.109 ± 0.012	0.085 ± 0.018
Entertainment	**0.267 ± 0.011✓**	0.173 ± 0.007	0.220 ± 0.011	0.188 ± 0.011
Health	**0.502 ± 0.010✓**	0.410 ± 0.017	0.397 ± 0.019	0.423 ± 0.020
Recreation	**0.235 ± 0.014✓**	0.045 ± 0.004	0.111 ± 0.011	0.119 ± 0.008
Reference	**0.387 ± 0.015✓**	0.360 ± 0.010	0.352 ± 0.009	0.350 ± 0.013
Science	**0.130 ± 0.011✓**	0.075 ± 0.007	0.064 ± 0.010	0.070 ± 0.017
Social	**0.533 ± 0.015✓**	0.340 ± 0.021	0.471 ± 0.014	0.449 ± 0.025
Society	**0.357 ± 0.043✓**	0.290 ± 0.019	0.254 ± 0.012	0.211 ± 0.041
Avg. Rank	**1.00✓**	3.19	2.75	3.06

**Table 7:** Comparison results of four compared methods in terms of subset accuracy for MLNB (The highest performance is shown in bold font and indicated by a check mark).

Dataset	Proposed	EGA+CDM	bALO-QR	CSO
RCV1 (S1)	**0.017 ± 0.007✓**	0.009 ± 0.002	0.012 ± 0.007	0.012 ± 0.005
RCV1 (S2)	**0.099 ± 0.012✓**	0.011 ± 0.005	0.087 ± 0.010	0.087 ± 0.004
RCV1 (S3)	**0.115 ± 0.018✓**	0.025 ± 0.005	0.093 ± 0.009	0.102 ± 0.005
RCV1 (S4)	**0.150 ± 0.008✓**	0.033 ± 0.014	0.120 ± 0.016	0.126 ± 0.016
RCV1 (S5)	**0.094 ± 0.014✓**	0.013 ± 0.003	0.082 ± 0.012	0.091 ± 0.011
Arts	**0.151 ± 0.010✓**	0.058 ± 0.007	0.071 ± 0.007	0.075 ± 0.006
Business	**0.527 ± 0.014✓**	0.514 ± 0.016	0.507 ± 0.011	0.512 ± 0.011
Computers	**0.351 ± 0.011✓**	0.299 ± 0.011	0.316 ± 0.010	0.319 ± 0.009
Education	**0.094 ± 0.011✓**	0.047 ± 0.009	0.074 ± 0.007	0.064 ± 0.013
Entertainment	**0.228 ± 0.010✓**	0.130 ± 0.009	0.188 ± 0.010	0.176 ± 0.022
Health	**0.389 ± 0.010✓**	0.307 ± 0.016	0.314 ± 0.009	0.308 ± 0.054
Recreation	**0.192 ± 0.010✓**	0.020 ± 0.003	0.093 ± 0.013	0.106 ± 0.016
Reference	**0.345 ± 0.011✓**	0.321 ± 0.006	0.316 ± 0.011	0.294 ± 0.074
Science	**0.109 ± 0.014✓**	0.053 ± 0.008	0.048 ± 0.005	0.055 ± 0.011
Social	**0.488 ± 0.016✓**	0.287 ± 0.022	0.432 ± 0.016	0.412 ± 0.012
Society	**0.284 ± 0.015✓**	0.215 ± 0.012	0.179 ± 0.028	0.157 ± 0.021
Avg. Rank	**1.00✓**	3.56	2.81	2.63

**Table 8:** Comparison results of four compared methods in terms of Hamming loss for ML-ELM (The highest performance is shown in bold font and indicated by a check mark).

Dataset	Proposed	EGA+CDM	bALO-QR	CSO
RCV1 (S1)	**0.037 ± 0.002✓**	0.038 ± 0.001	0.039 ± 0.001	0.040 ± 0.001
RCV1 (S2)	**0.034 ± 0.002✓**	0.037 ± 0.003	0.037 ± 0.001	0.036 ± 0.000
RCV1 (S3)	**0.033 ± 0.002✓**	0.037 ± 0.001	0.037 ± 0.003	0.036 ± 0.001
RCV1 (S4)	**0.033 ± 0.002✓**	0.036 ± 0.002	0.035 ± 0.001	0.034 ± 0.001
RCV1 (S5)	**0.034 ± 0.001✓**	0.036 ± 0.001	0.035 ± 0.001	0.035 ± 0.001
Arts	**0.080 ± 0.002✓**	0.092 ± 0.005	0.089 ± 0.001	0.088 ± 0.002
Business	**0.028 ± 0.001✓**	0.029 ± 0.001	0.029 ± 0.001	0.029 ± 0.001
Computers	**0.042 ± 0.001✓**	0.045 ± 0.001	0.044 ± 0.001	0.044 ± 0.001
Education	**0.052 ± 0.001✓**	0.060 ± 0.002	0.057 ± 0.001	0.056 ± 0.001
Entertainment	**0.078 ± 0.004✓**	0.088 ± 0.003	0.088 ± 0.004	0.083 ± 0.002
Health	**0.038 ± 0.001✓**	0.049 ± 0.002	0.047 ± 0.002	0.046 ± 0.001
Recreation	**0.090 ± 0.003✓**	0.115 ± 0.006	0.102 ± 0.003	0.100 ± 0.005
Reference	**0.034 ± 0.001✓**	0.038 ± 0.001	0.037 ± 0.001	0.037 ± 0.001
Science	**0.047 ± 0.003✓**	0.053 ± 0.002	0.051 ± 0.001	0.050 ± 0.001
Social	**0.026 ± 0.001✓**	0.036 ± 0.001	0.028 ± 0.001	0.029 ± 0.001
Society	**0.060 ± 0.001✓**	0.064 ± 0.002	0.062 ± 0.001	0.062 ± 0.001
Avg. Rank	**1.00✓**	3.75	2.88	2.38

**Table 9:** Comparison results of four compared methods in terms of one-error for ML-ELM (The highest performance is shown in bold font and indicated by a check mark).

Dataset	Proposed	EGA+CDM	bALO-QR	CSO
RCV1 (S1)	**0.531 ± 0.016✓**	0.704 ± 0.026	0.602 ± 0.018	0.614 ± 0.014
RCV1 (S2)	**0.526 ± 0.009✓**	0.715 ± 0.023	0.612 ± 0.017	0.611 ± 0.017
RCV1 (S3)	**0.521 ± 0.018✓**	0.727 ± 0.010	0.598 ± 0.020	0.606 ± 0.014
RCV1 (S4)	**0.484 ± 0.025✓**	0.698 ± 0.011	0.589 ± 0.020	0.567 ± 0.018
RCV1 (S5)	**0.512 ± 0.030✓**	0.692 ± 0.014	0.580 ± 0.025	0.588 ± 0.029
Arts	**0.542 ± 0.011✓**	0.633 ± 0.021	0.637 ± 0.018	0.626 ± 0.019
Business	**0.131 ± 0.008✓**	0.132 ± 0.007	0.133 ± 0.006	0.131 ± 0.007
Computers	**0.416 ± 0.010✓**	0.455 ± 0.009	0.441 ± 0.006	0.439 ± 0.009
Education	**0.594 ± 0.012✓**	0.636 ± 0.014	0.598 ± 0.013	0.620 ± 0.020
Entertainment	**0.527 ± 0.019✓**	0.591 ± 0.016	0.556 ± 0.019	0.569 ± 0.022
Health	**0.326 ± 0.014✓**	0.433 ± 0.017	0.422 ± 0.017	0.398 ± 0.023
Recreation	**0.541 ± 0.018✓**	0.741 ± 0.025	0.661 ± 0.019	0.666 ± 0.021
Reference	**0.450 ± 0.018✓**	0.511 ± 0.017	0.507 ± 0.014	0.502 ± 0.012
Science	**0.582 ± 0.018✓**	0.689 ± 0.025	0.663 ± 0.016	0.674 ± 0.021
Social	**0.355 ± 0.014✓**	0.512 ± 0.021	0.386 ± 0.017	0.421 ± 0.020
Society	**0.433 ± 0.011✓**	0.479 ± 0.018	0.470 ± 0.014	0.463 ± 0.015
Avg. Rank	**1.00✓**	3.88	2.63	2.50

**Table 10:** Comparison results of four compared methods in terms of Multi-label accuracy for ML-ELM (The highest performance is shown in bold font and indicated by a check mark).

Dataset	Proposed	EGA+CDM	bALO-QR	CSO
RCV1 (S1)	**0.275 ± 0.009✓**	0.214 ± 0.007	0.220 ± 0.006	0.215 ± 0.011
RCV1 (S2)	**0.305 ± 0.014✓**	0.198 ± 0.010	0.242 ± 0.016	0.243 ± 0.013
RCV1 (S3)	**0.320 ± 0.020✓**	0.202 ± 0.006	0.251 ± 0.010	0.258 ± 0.010
RCV1 (S4)	**0.343 ± 0.014✓**	0.215 ± 0.009	0.266 ± 0.009	0.275 ± 0.010
RCV1 (S5)	**0.309 ± 0.013✓**	0.206 ± 0.006	0.256 ± 0.012	0.256 ± 0.012
Arts	**0.362 ± 0.009✓**	0.275 ± 0.012	0.283 ± 0.009	0.284 ± 0.014
Business	0.686 ± 0.007	**0.686 ± 0.010✓**	0.680 ± 0.010	0.681 ± 0.008
Computers	**0.475 ± 0.008✓**	0.427 ± 0.010	0.441 ± 0.010	0.441 ± 0.008
Education	**0.337 ± 0.009✓**	0.286 ± 0.011	0.315 ± 0.012	0.318 ± 0.013
Entertainment	**0.418 ± 0.018✓**	0.336 ± 0.015	0.362 ± 0.014	0.362 ± 0.009
Health	**0.545 ± 0.013✓**	0.449 ± 0.011	0.462 ± 0.019	0.466 ± 0.012
Recreation	**0.379 ± 0.009✓**	0.210 ± 0.007	0.263 ± 0.017	0.285 ± 0.023
Reference	**0.493 ± 0.012✓**	0.437 ± 0.007	0.437 ± 0.016	0.447 ± 0.009
Science	**0.340 ± 0.017✓**	0.246 ± 0.011	0.254 ± 0.017	0.270 ± 0.014
Social	**0.583 ± 0.016✓**	0.435 ± 0.021	0.543 ± 0.015	0.519 ± 0.021
Society	**0.422 ± 0.014✓**	0.392 ± 0.010	0.398 ± 0.011	0.402 ± 0.011
Avg. Rank	**1.06✓**	3.81	2.81	2.31

**Table 11:** Comparison results of four compared methods in terms of subset accuracy for ML-ELM (The highest performance is shown in bold font and indicated by a check mark).

Dataset	Proposed	EGA+CDM	bALO-QR	CSO
RCV1 (S1)	**0.025 ± 0.016✓**	0.012 ± 0.002	0.011 ± 0.006	0.013 ± 0.006
RCV1 (S2)	**0.114 ± 0.009✓**	0.011 ± 0.003	0.090 ± 0.012	0.099 ± 0.009
RCV1 (S3)	**0.129 ± 0.017✓**	0.011 ± 0.004	0.108 ± 0.007	0.111 ± 0.009
RCV1 (S4)	**0.166 ± 0.016✓**	0.023 ± 0.005	0.120 ± 0.014	0.126 ± 0.012
RCV1 (S5)	**0.113 ± 0.009✓**	0.008 ± 0.003	0.090 ± 0.014	0.092 ± 0.012
Arts	**0.190 ± 0.011✓**	0.118 ± 0.009	0.143 ± 0.009	0.140 ± 0.020
Business	0.528 ± 0.008	0.527 ± 0.015	0.526 ± 0.013	**0.529 ± 0.011✓**
Computers	**0.372 ± 0.010✓**	0.323 ± 0.011	0.338 ± 0.011	0.340 ± 0.007
Education	**0.247 ± 0.009✓**	0.186 ± 0.016	0.197 ± 0.019	0.214 ± 0.010
Entertainment	**0.326 ± 0.018✓**	0.231 ± 0.021	0.243 ± 0.017	0.276 ± 0.013
Health	**0.408 ± 0.014✓**	0.315 ± 0.015	0.325 ± 0.011	0.352 ± 0.014
Recreation	**0.270 ± 0.041✓**	0.086 ± 0.010	0.137 ± 0.016	0.146 ± 0.017
Reference	**0.427 ± 0.015✓**	0.376 ± 0.007	0.379 ± 0.013	0.386 ± 0.015
Science	**0.228 ± 0.030✓**	0.153 ± 0.015	0.176 ± 0.015	0.179 ± 0.013
Social	**0.520 ± 0.011✓**	0.333 ± 0.018	0.482 ± 0.017	0.468 ± 0.014
Society	**0.296 ± 0.014✓**	0.274 ± 0.012	0.281 ± 0.010	0.289 ± 0.014
Avg. Rank	**1.06✓**	3.88	3.00	2.06

**Table 12:** Friedman statistics and critical value in terms of each evaluation measure for MLNB.

Evaluation Measure	Friedman Statistics	Critical Values (α=0.05)
Hamming loss	101.914	2.812
One-error	63.304	
Multi-label accuracy	24.520	
Subset accuracy	34.557	

**Table 13:** Friedman statistics and critical value in terms of each evaluation measure for ML-ELM.

Evaluation Measure	Friedman Statistics	Critical values (α=0.05)
Hamming loss	61.632	2.812
One-error	81.314	
Multi-label accuracy	51.484	
Subset accuracy	114.668	
